# Exploring metabolic syndrome care: insights from community pharmacists in the UAE setting-a cross-sectional questionnaire-based study

**DOI:** 10.3389/fpubh.2026.1748459

**Published:** 2026-02-16

**Authors:** Ammar Abdulrahman Jairoun, Moyad Shahwan, Abeer M. Al-Ghananeem, Sabaa Al Hemyari, G. R. H. Alnuaimi, Manar Al Kazhali, Ammar Ali Saleh Jaber

**Affiliations:** 1 Health and Safety Department, Dubai Municipality, Dubai, United Arab Emirates; 2 Discipline of Clinical Pharmacy, School of Pharmaceutical Sciences, Universiti Sains Malaysia (USM), Pulau Pinang, Malaysia; 3 Centre of Medical and Bio-allied Health Sciences Research, Ajman University, Ajman, United Arab Emirates; 4 Center of Exellence in Precision Medicine and Digital Health, Department of Physiology, Faculty of Dentistry, Chulalongkorn University, Bangkok, Thailand; 5 College of Pharmacy and Health Sciences, Ajman University, Ajman, United Arab Emirates; 6 Pharmacy Department, Emirates Health Services, Dubai, United Arab Emirates; 7 Higher Colleges of Technology, Health Sciences, Dubai, United Arab Emirates

**Keywords:** attitude, community pharmacist, metabolic syndrome, perception, practice, prevention

## Abstract

**Background:**

Community pharmacists act as a middleman among both patients and prescribing doctors. As such, they oversee making sure that patients receive the best possible pharmaceutical care for Metabolic syndrome (MetS). There is a dearth of adequate data on MetS awareness, attitudes, and treatment practices between community pharmacists within the UAE.

**Objectives:**

To assess the attitudes of community pharmacies professionals across Abu Dhabi, Dubai, and the Northern Emirates toward managing and preventing MetS.

**Methods and materials:**

This is a cross-sectional study design carried out between May 2023 and February 2024. Four final-year pharmacy students conducted in-person interviews among professionals working in community pharmacies and structured questionnaire was utilized for information collection. The questionnaire comprised Demographic information, attitudes and practices to Prevention and Management of Metabolic Syndrome.

**Results:**

The total of 420 pharmacists were recruited for the study. The mean attitude score toward the prevention and treatment of metabolic syndrome was 75.8%, with a 95% confidence interval (CI) of [76.3, 81.4%]. The average practice score toward prevention and treatment of metabolic syndrome was 86.8% with a 95% CI of [85.3, 88.3%]. Supervising pharmacists were more likely to achieve a positive attitude score compared with assistant pharmacists (OR = 4.63; 95% CI: 2.32–9.21; *p* < 0.001). Pharmacists with more than 10 years of experience had higher odds of a positive attitude score compared with those with 1–5 years of practice (OR = 2.11; 95% CI: 1.11–3.99; *p* = 0.022). Good practice scores were significantly associated with working in chain pharmacies (OR = 1.69), holding senior professional positions (supervising pharmacist: OR = 8.39; chief pharmacist: OR = 5.90), having longer professional experience (6–10 years: OR = 5.08; >10 years: OR = 7.04), and receiving prior training on MetS (OR = 4.64; 95% CI: 2.52–8.55; *p* < 0.001).

**Conclusion:**

The findings show that the community pharmacists are cognizant about appropriate practices required for the treatment and management of MetS. It was inferred that the setting and quality (experience and training) of pharmacy professionals play vital roles in strengthening the attitude and practice of community pharmacists with regards to MetS.

## 1 Introduction

Metabolic disorder (MetS) involves a cluster or group of comorbid health conditions that when combined increase the hazard of cardiovascular disease, stroke (1). MetS is defined as comorbidity of obesity, hypertension, Type 2 diabetes, abnormal cholesterol levels and disturbances in carbohydrate and lipid metabolism. In the presence of concurrent conditions they increase the risk of developing organ damage, atherosclerosis and coronary artery disease (2). Twenty to 25 % of adults worldwide are thought to have MetS, although each condition is treatable with lifestyle changes and/or medication. Owing to its relationship between Type 2 diabetes (T2DM) and cardiovascular disease, both mortality and quality of life are impacted by MetS (3). Eating high calorie foods, inactive lifestyle, smoking, and the utilize of antiretroviral medications have all been recognized as main risk factors related to the rise in MetS (4).

The primary goals of treating MetS are to reduce the risk of heart disease and prevent type 2 diabetes. Patients with metabolic syndrome are five times more likely to develop type 2 diabetes. Consequently, they face twice the risk of mortality and three times the risk of experiencing a heart attack or stroke compared to individuals without metabolic syndrome (5). In cases where Type 2 diabetes already exists, treatment can lower the risk of heart disease through the control of all risk factors. Management of MetS usually includes losing weight and becoming more active.

Attention and efforts are now constantly given to prevention and reduction of this T2DM (3). One key strategy for preventing type 2 diabetes is the effective treatment and managing of metabolic syndrome. This highlights the need to increase awareness and understanding of metabolic syndrome, a condition that remains poorly understood despite its complexity. The rise in obesity, the progression of hypertension, and the worsening of metabolic disorders contribute to the development of additional diseases, further raising cardiovascular risk (6).

General practitioners have the responsibility for caring for general patients and therefore must have adequate knowledge of MetS, a positive attitude and good medical practices (7). Nevertheless, as health professionals are among the most accessible and well-positioned health professionals in the community, they could play a critical role in MetS prevention and treatment efforts (8). This can also be explained by the fact that community drug retail outlets where community pharmacists operate provide a platform for active interaction with host communities. This is why it is imperative that community pharmacists are aware and knowledgeable about MetS.

A number of studies have evaluated MetS awareness or CVD-related knowledge. Different results have been recorded. For instance, while Nadas et al. (9) and See et al. (10) reported 12.5 to 29.0% of their study participants are aware about MetS, respectively. Lewis et al. (11) noted that only ≥10 of its participants comprehended the definition of MetS. Alshuniefi et al. (7) reported in a study conducted in Riyadh, Saudi Arabia, that primary care residents had a low level of knowledge about MetS, although more than 50 % of them had positive attitudes and half demonstrated good practices. Katoue et al. (12) examined the role of community pharmacists in the prevention and treatment of metabolic syndrome in Kuwait. The study found significant gaps in local pharmacists’ awareness of MetS.

MetS is a growing challenge in the United Arab Emirates (UAE) (13). Furthermore, the frequency of overweight and obesity is increasing sharply in the UAE. Despite this issue, there is a need of evidence regarding the awareness, knowledge and practices of community pharmacists in the UAE concerning the risk factors associated with MetS. In one focus group discussion with nine pharmacists, Alozaibi (14) reported that not all pharmacists were familiar with the MetS concept or definition. Alozaibi earlier identified the role of hospital pharmacists in the UAE in managing patients with MetS as well as factors influencing pharmacists’ involvement in their care in one of Abu Dhabi foremost hospitals. The study revealed that the pharmacists were disillusioned by the limited utilization of their knowledge and communication skills despite their high accessibility (14).

Evidence points to an expanded role for pharmacists across community and clinical settings in the management of chronic disease. Studies from the Middle East show that community pharmacists increasingly participate in public health activities such as lifestyle counselling, prevention, and patient education, particularly for obesity and asthma (15, 16). Pharmacists commonly recognise these conditions as major public health concerns and report advising patients on diet, physical activity, and medication use.

Effectiveness remains constrained by system-level limitations. Gaps in formal training, lack of structured clinical tools, limited consultation time, insufficient staffing, absence of private counselling areas, and no reimbursement for extended services are reported across practice settings (15, 16). These constraints reduce the consistency and depth of pharmacist-led interventions.

In clinical and hospital settings, the pattern differs. When pharmacists are embedded within multidisciplinary teams, structured involvement is associated with improved clinical outcomes, including better glycaemic control and fewer treatment-related complications among high-risk patients (17). Emerging work also highlights pharmacists’ role in early identification and referral for chronic metabolic and liver-related conditions, reflecting their accessibility and continuity of patient contact (18). Together, these findings support systematic evaluation of pharmacists’ attitudes, practices, and readiness across roles, alongside targeted training, supportive policy frameworks, and integrated care models.

There is insufficient data on MetS awareness, attitudes and treatment practices among community pharmacists in the UAE. Therefore, this study examines the attitudes of community pharmacy professionals working in Abu Dhabi, Dubai and the Northern Emirates toward the treatment and prevention of metabolic syndrome. Respondents’ practices and strategies for treating and preventing metabolic syndrome were also assessed.

## 2 Methods and materials

### 2.1 Study setting and design

The aim of this cross-sectional research was to evaluate the perspectives and practices of community pharmacists regarding the treatment and prevention of metabolic syndrome in the United Arab Emirates. Between May 2023 and February 2024, four final year pharmacy students conducted surveys among community pharmacy professionals working in Abu Dhabi, Dubai and the Northern Emirates. Before conducting the face-to-face interviews, students received comprehensive training on questionnaire administration and communication using the scientific terms used in the study. The training, which was derived from prior experiences, highlighted the importance of planning, preparation, and training in enhancing interviewer competency and reducing data collection errors.

### 2.2 Research instrument development, including pilot testing

Based on the literature review, a structured questionnaire was designed (8) and then adapted to the context of the UAE by taking into consideration the most relevant research themes. The proposed questionnaire was tested by experts in pharmacy practice, diabetology, and metabolic syndrome for its appropriateness. Secondly, five members from the Faculty of Medicine and Clinical Pharmacy at Ajman University were also asked about the suitability and relevance of this questionnaire. Minor modifications, which included defining scientific terms and modifying question numbering, were made as per their recommendations prior to piloting. The content validity was established using the method by Lawshe (19), where a Content Validity Ratio (CVR) of more than 0.78 was deemed acceptable (19, 20). Minor modifications, which included defining scientific terms and modifying question numbering, were made as per their recommendations prior to piloting. All the items in the questionnaire had values higher than this threshold and, therefore, possessed enough validity. Then, the Content Validity Index (CVI) was determined, which turned out to be 0.881, satisfying the threshold value, hence confirming overall validity (20).

The questionnaire was subjected to a pilot test from April 14 to April 28, 2023, on a sample size of fifty conveniently sampled community pharmacists. Their responses were excluded in the final analysis, and forty fully completed the survey. Then, the reliability of the questionnaire and the required sample size for the main study were estimated according to the outcomes of the pilot study.

Reliability assessment of the questionnaire using Cronbach’s *α* yielded an acceptable value of 0.75 for internal consistency (20). These steps ascertained the validity and reliability of the questionnaire to provide a sound foundation for the investigation.

### 2.3 Research instrument sections

The study questionnaire consisted of three main sections:

#### 2.3.1 Part 1: Demographic information (5 questions)

- Gender
- Years of experience working as a pharmacist in any capacity, including roles such as chief pharmacist or pharmacist in charge
- Total years of professional experience
- Completion of courses related to the management and prevention of metabolic syndrome

#### 2.3.2 Part 2: Attitudes toward managing and preventing metabolic syndrome (5 questions)

- Designed to gauge respondents’ opinions and attitudes toward managing and preventing metabolic syndrome

#### 2.3.3 Part 3: Practices used to manage and prevent metabolic syndrome

- These sections contained 14 questions specifically targeting respondents’ approaches, strategies, and practices related to managing and preventing metabolic syndrome (The full questionnaire used in this study is provided as Supplementary file 1).

### 2.4 Questionnaire scoring

Respondents’ attitudes toward treatment and prevention of metabolic syndrome were rated as “Agree,” “Neutral,” or “Disagree.” In line with their overall tone, these responses were categorized as “positive attitude” or “negative attitude.” Positive attitude responses were allocated one point, while negative attitude responses were allocated zero points. The total raw score of each respondent was calculated by summing the scores for all five items.

To assess the strategies of the respondents on how to manage and prevent metabolic syndrome, a categorical response of “yes” or “no” was used. For every “yes,” one score was given, while every “no” was given a score of zero; thus, the highest score possible for this category is 14.

The attitude and practice proportions for each respondent were then calculated, ranging from 0 to 100%, to provide information on the general attitudes and practices of community pharmacists regarding the treatment and prevention of metabolic syndrome.

Attitude and practice were categorized using median-based thresholds, a standard approach in cross-sectional survey analysis. The attitude domain comprised five items (score range: 0–5) with a median of 4; scores of 4 or higher were classified as a positive attitude. The practice domain included fourteen items (score range: 0–14) with a median of 13; scores of 13 or higher were classified as good practice.

### 2.5 Sample size calculation and target population

In determining the sample size of the main study, results were pegged on an overall response rate observed at 80% during the pilot study. To reach an estimation of the final sample size, the respondents were asked if they had previous experience in the prevention and treatment of metabolic syndrome, to which approximately half responded yes.

Assuming a 5% alpha level, a 95% CI width of 10% as determined by a precision (D) setting of 5%, and a possible non-response rate of about 10%, the proposed sample size was sufficient under these assumptions of 426 respondents.

The criteria for selecting the sample of the study included community pharmacists who are at least three months into their professional experience, working independently or for registered chains regulated by authorities like the Ministry of Health, the Health Authority Abu Dhabi (HAAD), or the Dubai Health Authority. The exclusion criteria that was applied was as follows:

- Pharmacists with less than three months of professional experience (including new recruits or those on probation)
- Pharmacists not registered with the authorities identified above.

### 2.6 Sampling technique

A random sampling approach was employed to ensure the sample was representative of the true population of almost 1,300 licensed community pharmacists and 2000 active community pharmacies in the UAE (21–23). The contact details of community pharmacies were obtained from local business directories and the Yellow Pages.

To ensure the sample was representative of community pharmacies in the different UAE regions, a stratification sampling technique was based on the locations of operational community pharmacies, with three strata: Dubai, Abu Dhabi, and the Northern Emirates.

Important data related to the selected community pharmacies was noted in an Excel spreadsheet after stratification, which was a sample frame. This data included pharmacist name, location, type, telephone number, and email address. Each pharmacy was assigned an ID number for identification. In total, 426 community pharmacies were selected from the sample Excel frame using simple random sample selection. The selected community pharmacies were subsequently arranged by type and their location within the UAE:

This systematic sampling approach was designed to ensure a proper representation of community pharmacies from various regions in the UAE with the underlying intention of generating findings that were generalizable to the wider population of community pharmacists.

### 2.7 Data collection

Visits to the selected community pharmacies in Abu Dhabi, Dubai, and the Northern Emirates took place from May 15, 2023, to February 22, 2024. The goal of this study was explained to the pharmacists, and their cooperation was requested by providing their email addresses for this purpose. The structured questionnaire was subsequently administered through in-person interviews with the help of experienced researchers.

### 2.8 Statistical analyses

Data analyses were performed using SPSS Version 26. Quantitative variables were summarized as mean ± standard deviation (±SD) if normally distributed. Categorical variables were presented as frequencies and percentages. The median and IQR were used as summary statistics for continuous skewed data. Quantitative variables between groups were compared using appropriate statistical tests, including one-way ANOVA and non-parametric alternatives and unpaired Student’s t-tests.

In addition, multivariate logistic regression models were used to identify factors affecting the attitudes, knowledge, and behaviors of the respondents with regard to the prevention and management of metabolic syndrome. The level of statistical significance was assumed with a *p*-value below 0.05, using this as a guideline in interpreting the results. Full analysis was therefore carried out to gauge various factors influencing the surveyed respondents’ perceptions and practices on metabolic syndrome.

### 2.9 Ethical considerations

The study received ethical approval from the Institutional Ethical Review Committee of Ajman University (P-H-S-2023-2-13). After explaining the goals of the study, prior to data collection, all pharmacists were assured that participation was voluntary. Written informed consent was obtained from all pharmacists to ensure that they agreed to answer in the questionnaire. No record of the pharmacists’ identities was kept in any way throughout the study, and their confidentiality was guaranteed.

## 3 Results

### 3.1 Demographic information

The study recruited 420 pharmacists in total. Among them, more than half were female (69.5%), while a third was male (30.5%). Regarding experience, 70 pharmacists (16.7%) had 1–5 years of experience, 182 (43.3%) had 6–10 years of experience, and 168 (40%) had > 10 years of experience. Chain pharmacists comprised 53.8% of the subjects, while independent pharmacies made up 46.2%. In terms of pharmacist positions, 40% were Pharmacist in Charge, 42.4% were Chief Pharmacists, and 17.1% were pharmacy assistants. Furthermore, the majority of study pharmacists completed a training on the preventing and management of MetS (78.1%) (Table 1).

**Table 1:** Demographic information (*n* = 420).

Demographic	Groups	Number	Percentage
Gender	Female	292	69.5
	Male	128	30.5
Years in practice	1 to 5 Years	70	16.7
	6–10 Years	182	43.3
	>10 Years	168	40
Type of pharmacy	Privately held pharmacy	194	46.2
	Retail pharmacy	226	53.8
Pharmacist position	Supervising pharmacist	170	40.5
	Chief pharmacist	178	42.4
	Assistant pharmacist	72	17.1
Received training in the prevention and management of MetS.	Yes	328	78.1
	No	92	21.9

### 3.2 The attitudes and practices of community pharmacists regarding the prevention and treatment of metabolic syndrome

The average attitude score regarding prevention and treatment of metabolic syndrome was 75.8% with a 95% confidence interval (CI) of [76.3, 81.4%]. The average practice score toward prevention and treatment of metabolic syndrome was 86.8% with a 95% CI of [85.3, 88.3%].

Table 2 compares the attitude and practice scores based on demographics. A statistically significant association was found between pharmacist position and attitude score, with higher scores observed among Pharmacist in Charge and Chief Pharmacists compared to Assessment Pharmacists (*p* < 0.001). Similarly, years of experience showed a statistically significant association with attitude scores, indicating higher scores among pharmacists with more years of experience (*p* < 0.001).

**Table 2:** Comparison of attitude and practice scores based on demographic groups.

Demographics	Attitude score (5 items)				Practice score (14 items)			
	Mean %	95% CI		*P*-value	Mean %	95% CI		*P*-value
Gender								
Female	79.73	76.62	82.83	0.313	85.81	83.93	87.69	0.033
Male	76.87	72.34	81.40		89.28	87.06	91.51	
Type of pharmacy								
Privately held pharmacy	78.97	74.88	83.05	0.936	82.76	80.13	85.40	<0.001*
Chain Pharmacy	78.76	75.53	81.98		90.39	88.96	91.81	
Position in the pharmacy								
Supervising pharmacist	86.11	82.44	89.78	<0.001*	94.45	92.99	95.92	<0.001*
Chief pharmacist	76.63	72.90	80.35		84.83	82.54	87.11	
Assistant pharmacist	67.22	60.06	74.38		74.01	70.16	77.85	
Experiences								
1–5 Years	65.14	57.07	73.21	<0.001*	71.22	66.43	76.01	<0.001*
6–10 Years	80	76.74	83.25		87.12	85.26	88.98	
> 10 years	83.33	79.39	87.27		93.11	91.50	94.72	
Trained on prevention of metabolic syndrome				0.342				
Yes	79.51	76.69	82.33		89.02	87.45	90.59	<0.001*
No	76.52	70.55	82.49		79.19	75.81	82.57	

*p-values less than 0.05 interpreted statistically significant, these values were obtained from independent t test and One Way ANOVA. Mean percentage score, derived from the raw scores.

In terms of the practice of preventing and managing metabolic syndrome, several factors were associated with better scores. These include male pharmacists (*p* = 0.033), chain pharmacies compared to independent pharmacies (*p* < 0.001), Pharmacist in Charge and Chief Pharmacists compared to Assessment Pharmacists (*p* < 0.001), pharmacists with more years of experience (*p* < 0.001), and those who received training on the prevention of metabolic syndrome (*p* < 0.001).

Tables 3, 4 displayed the answers to all of the questions pertaining to MetS knowledge, attitudes, and practices.

**Table 3:** Frequency and proportion of pharmacists’ responses regarding attitudes toward preventing and treating metabolic syndrome.

Attitude items	Disagree		Neutral		Agree	
	*F*	%	*F*	%	*F*	%
Early identification of patients with metabolic syndrome is crucial for addressing their diverse risk factors effectively.	26	6.2	48	11.4	346	82.4
There is a strong association between obesity and leading sedentary lifestyles, both of which are closely linked to metabolic syndrome.	28	6.7	54	12.9	338	80.5
There is a pressing need for significant changes in the habits of the general public in the United Arab Emirates.	28	6.7	54	12.9	338	80.5
The population in the United Arab Emirates is cognizant of the correlation between metabolic syndrome and an increased predisposition to cardiovascular diseases and other non-communicable diseases (NCDs).	38	9	54	12.9	328	78.1
Metabolic syndrome is prevalent in the UAE, and its incidence is on the rise.	66	15.7	48	11.4	306	72.9

F, frequency; %, Proportion.

**Table 4:** Frequency and proportion of pharmacists’ responses regarding practice toward preventing and treating metabolic syndrome.

Practice items	Yes		No	
	*F*	%	*F*	%
Advising hypertension patients to limit their salt intake.	386	91.9	34	8.1
Counseling patients on the benefits of consuming more soluble fiber.	386	91.9	34	8.1
Referring patients to appropriate healthcare professionals or clinics as needed	384	91.4	36	8.6
Encouraging patients to increase their physical activity levels.	380	90.5	40	9.5
Selling home blood pressure and glucose monitoring devices to patients.	380	90.5	40	9.5
Providing dietary advice aimed at lowering cholesterol levels by reducing intake of cholesterol and saturated fat.	378	90	42	10
Maintaining comprehensive records of all patient care services provided.	374	89	46	11
Recommending a low-calorie diet to aid in weight reduction for patients.	372	88.6	48	11.4
Educating patients about the importance of regular monitoring of blood pressure, glycemic status, and weight, emphasizing the significance of achieving desired health outcomes.	368	87.6	52	12.4
Counseling patients to adhere to their prescribed treatment regimens.	350	83.3	70	16.7
Monitoring and evaluating patient responses to treatment plans.	348	82.9	72	17.1
Advising patients on over-the-counter medications and self-care techniques for managing components of metabolic syndrome	344	81.9	76	18.1
Advising patients to eat more vegetables that are derived from plants.	342	81.4	78	18.6
Offering guidance and support to patients in quitting smoking	316	75.2	104	24.8

F, frequency; %, Proportion.

Table 5 displays the findings of the multivariate logistic regression analysis for factors associated with attitudes and practices toward the prevention and treatment of metabolic syndrome.

**Table 5:** Regression analysis identifying factors affecting pharmacists ‘attitude and practice on prevention and treatment of metabolic syndrome.

Demographics	Positive attitude ≥ 4				Good practice ≥ 13			
	OR	95% CI		*P*-value	OR	95% CI		*P*-value
Gender (Ref. Female)								
Male	0.630	0.400	1.992	0.071	1.609	0.927	2.792	0.091
Type of pharmacy (Ref. Privately held pharmacy)								
Chain pharmacy	1.553	0.363	1.842	0.232	1.687	1.014	2.809	0.044*
Position in the pharmacy (Ref. Assistant pharmacist)								
Supervising pharmacist	4.625	2.322	9.211	<0.001*	8.389	4.772	11.145	<0.001*
Chief pharmacist	1.706	0.908	3.204	0.097	5.899	2.515	8.840	<0.001*
Years in practice (Ref. 1–5 Years)								
6 to 10 Years	1.733	0.883	3.402	0.110	5.075	2.801	8.671	0.001*
> 10 years	2.107	1.114	3.986	0.022*	7.039	3.830	10.808	<0.001*
Training on metabolic syndrome (Ref. No)								
Yes	1.007	0.600	1.690	0.978	4.638	2.516	8.551	<0.001*

^*^P-values less than 0.05 interpreted statistically significant.

Good knowledge and positive attitude scores were determined based on median score.

Supervising pharmacists were more likely to achieve a positive attitude score compared with assistant pharmacists (OR = 4.63; 95% CI: 2.32–9.21; *p* < 0.001). Pharmacists with more than 10 years of experience had higher odds of a positive attitude score compared with those with 1–5 years of practice (OR = 2.11; 95% CI: 1.11–3.99; *p* = 0.022).

With regard to the type of pharmacy, Pharmacists employed in chain pharmacies were more likely to achieve a good practice score compared with those working in privately held pharmacies (OR = 1.69; 95% CI: 1.01–2.81; *p* = 0.044).

Professional position was strongly associated with practice scores. Supervising pharmacists had significantly higher odds of achieving a good practice score compared with assistant pharmacists (OR = 8.39; 95% CI: 4.77–11.15; *p* < 0.001). Similarly, chief pharmacists also demonstrated higher odds compared with assistant pharmacists (OR = 5.90; 95% CI: 2.52–8.84; *p* < 0.001).

Years of professional experience showed a significant association with practice scores. Pharmacists with 6–10 years of experience had higher odds of achieving a good practice score compared with those with 1–5 years of experience (OR = 5.08; 95% CI: 2.80–8.67; *p* = 0.001). The odds were further increased among pharmacists with more than 10 years of experience (OR = 7.04; 95% CI: 3.83–10.81; *p* < 0.001).

Previous training on metabolic syndrome was significantly associated with good practice scores. Pharmacists who reported receiving training had higher odds of achieving a good practice score compared with those without prior training (OR = 4.64; 95% CI: 2.52–8.55; *p* < 0.001).

## 4 Discussion

The wide-ranging impact of community pharmacists from their conventional dispensing role to significantly contributing to population health is being acknowledged globally (24). This study is the first survey to examine community pharmacists’ awareness, knowledge and practices in the prevention and treatment of MetS in the United Arab Emirates. The objective of the study was to explore the attitudes and practices of community pharmacists toward the prevention and treatment of metabolic syndrome in the UAE. The average attitude and practice scores regarding prevention and treatment of MetS were 75.8 and 86.8%, respectively. This suggests that professionals largely agreed with the basic points regarding MetS, were involved in advising MetS patients on prevention and management and recognized the effectiveness of key interventions for the prevention and treatment of MetS (8).

72.9% of the respondents agreed that MetS is prevalent in the UAE, with a growing incidence. Similarly, a study performed in United States reported that 75 and 61% of pharmacists strongly agreed that MetS diseases that include chronic heart disease and elevated blood cholesterol levels, respectively, are major health issues that face Americans (25). Also, a study conducted in Kuwait reported that 94.5% of community pharmacy professionals agreed that MetS is prevalent and rising in the country (12). Belachew et al. (8) revealed that majority of respondents in a study conducted in Ethiopia “agreed” and “strongly agreed” MetS is common and is a rising health issue. In addition, Via-Sosa et al. (26) reported pharmacists believed that premorbid MetS is rising at an alarming rate.

80.5% of the pharmacists agreed obesity and sedentary lifestyles are closely linked to MetS. This result is consistent with those of previous studies (8). This finding also concurs with the result of Katoue et al. (12), which reported that community pharmacists believed MetS as a prevalent health issue in the Kuwait that is mainly associated with obesity and sedentary lifestyles.

78.1% of the respondents agreed that UAE public is cognizant of the relationship between MetS and an elevated risk of cardiovascular diseases and other non-communicable diseases. This can be attributed to the fact that majority of the persons who patronize the retail outlets are familiar about MetS, with some knowledge about health-related issues due to the active presence of public awareness campaign events. In contrast, studies like that of Belachew et al. (8) reported that a high percentage of respondents in their study either disagreed (41.5%) or remained neutral (36.9%) about public awareness about the association between MetS and the high risk for cardio-vascular diseases, which could be due to poor education of the clients visiting retail outlets and lack public awareness campaigns.

This study showed that 82.4% agreed that early identification of patients with MetS is critical for effectively addressing their various risk factors. The effectiveness of the pharmacists’ role in the early identification of high-risk patients and subsequent referrals to physicians has been reported previously (27, 28).

Responses were elicited from the pharmacists on statements relating to practices that are associated with the prevention and management of metabolic syndrome. Most of the respondents agreed that diet control and physical activity formed the basis for addressing MetS. Previous studies have established that community pharmacists are well placed to offer weight management advice (12, 29). The respondents agreed that patients with MetS should be advised to limit salt intake, increase soluble fiber consumption, and decrease cholesterol levels. The results of these studies suggest that community pharmacists represent appropriate and accessible channels through which to deliver education on MetS control practices (8).

Regarding smoking, 75.2% of the respondents agreed that patients should be provided with advice and support to quit smoking. Community pharmacists are well-positioned to advise on quitting smoking (12, 30). These findings disagree with the results by Belachew et al. (8), in which 49.2% of the respondents advised MetS patients about quitting smoking. Additionally, most of the respondents in the present study advised patients regarding the benefits of weight control, management of blood pressure, and glucose measurement. These findings agree with the results of several studies (8, 12).

Above 80% of the respondents agreed that the patients should be given advice and help to enable them to carry out their treatment in line with the prescription, and their response to treatment plans should be monitored and evaluated.

These findings are analogous with the results of other studies performed in USA (25), Kuwait (12) and Ethiopia (8). This finding suggests that patient care documentation systems should be a component of community pharmacies to enhance the evaluation of patients’ responses and improve quality of services. Given the proper education and training, community pharmacists could be important front-line contributors to the control of this emerging epidemic in UAE. Most of the community pharmacists in this study were involved in encouraging patients’ adherence with prescribed treatments, monitoring patients’ response to therapy, and advising patients on over-the-counter medications and self-care techniques for managing components of MetS. This indicates significant awareness among community pharmacists with regards to MetS. Thus, the community pharmacists should be front-line contributors to the control of MetS in the UAE.

Furthermore, >80% of the respondents advised patients to eat more vegetables that are derived from plants. This perceived effectiveness of using herbals is consisted with related studies (8); although other studies have argued that lifestyle interventions were more effective for MetS treatment and management than herbals (31).

This study also managed to analyze the association of socio-demographic characteristics with attitude and practice of the community pharmacists with regards to MetS. A better attitude toward prevention and treatment of metabolic syndrome was observed among ‘Pharmacists in Charge’ and pharmacists with more than 10 years of experience. Years of experience showed a statistically significant association with attitude scores. In terms of the practice of preventing and managing MetS, chain pharmacies, Pharmacist in Charge and Chief Pharmacists, pharmacists with more years of experience, and those who received training on the prevention of MetS were associated with better scores based on the multivariate logistic regression analysis. Thus, it can be inferred that the setting and quality (experience and training) of pharmacy professionals play vital roles in strengthening the attitude and practice of community pharmacists with regards to the treatment and management of MetS.

Community pharmacy practice is shaped by limited access to formal training, time pressure, absence of private counselling space, and lack of reimbursement for extended patient care. In settings with minimal institutional support, pharmacists face difficulty converting knowledge into routine practice despite strong motivation.

The association between prior training and higher practice scores points to the value of structured educational programmes that prioritise practical skills, workflow integration, and patient communication. Strengthening practice capacity therefore requires system-level support alongside individual competence. Further studies based on prospective or mixed-methods approaches is needed to clarify how training, pharmacy ownership, gender, and workplace resources work in combination to shape practice behaviour. Stratified analyses, interaction modelling, and qualitative methodologies would help delineate subgroup-specific barriers and guide targeted training and policy interventions to optimise pharmacists’ role in chronic disease management and prevention.

Overall, the findings of this study show that community pharmacists are cognizant about appropriate practices required for the treatment and management of MetS, based on consistency of the results with similar studies. Therefore, effective therapeutic and management programs need to be designed and implemented to empower community pharmacists to provide efficacious pharmaceutical care services to MetS patients.

Despite the strengths of the work, it is constrained by some limitations that should be considered in the course of interpreting the results. Since the study was only conducted in Abu Dhabi and northern emirates, caution should be prudently exercised when generalizing to other cities and regions in UAE. In addition, the information provided by pharmacists may be subject to personal perceptions; hence the verifying the accuracy or truthfulness of the responses is not possible. Also, the responses of the pharmacists depend on their capacity to recollect experiences with patients. Moreover, the visit of the pharmacy students to community pharmacists at their workplace to provide them the questionnaire could create respondent bias, which could have been avoided if the study was clinical or a simulated pharmacist-patient setting.

## 5 Conclusion

The attitude and practice community pharmacists in the UAE with regards to the treatment and management of MetS patients were evaluated. The overall contribution of the health professionals in counseling patients, providing valid opinions about MetS, and their perception toward the effectiveness of interventions was found to be positive. The community pharmacists indicated that they are able to offer valuable counsel to MetS patients about the syndrome, as well as the provision of services, such as evaluation and monitoring patients’ responses to treatment. The findings show that the community pharmacists are cognizant about appropriate practices required for the treatment and management of MetS. It was inferred that the setting and quality (experience and training) of pharmacy professionals play vital roles in strengthening the attitude and practice of community pharmacists with regards to MetS.

## Data Availability

The datasets generated and/or analyzed during the current study are not publicly available due to ethical and confidentiality considerations. Requests to access these datasets should be directed to corresponding authors.
