# Supplementary material for: Exploring metabolic syndrome care: insights from community pharmacists in the UAE setting-a cross-sectional questionnaire-based study
Source: Front Public Health. 2026 Feb 16;14:1748459. doi: 10.3389/fpubh.2026.1748459 (PMC12950765; doi:10.3389/fpubh.2026.1748459)
Supplement: Supplementary file 1 [file Data_Sheet_1.pdf]

METABOLIC SYNDROME ATTITUDE AND PRACTICE AMONG COMMUNITY PHARMACISTS

Study Questionnaire

Contents

Section 1: demographic information 2

Section 2: Attitudes regarding the prevention and treatment of metabolic syndrome. 3

Section 3: Practices regarding the prevention and treatment of metabolic syndrome. 4

Section 1: demographic information

**Demographic**	Response
**Gender**	☐ Female
	☐ Male
**Years in practice**	☐ 1 to 5 Years
	☐ 6-10 Years
	☐ > 10 Years
**Type of pharmacy**	☐ Privately held pharmacy
	☐ Retail pharmacy
**Pharmacist position**	☐ Supervising Pharmacist
	☐ Chief pharmacist
	☐ Assistant pharmacist
**Received training in the prevention and management of Metabolic syndrome**	☐ Yes
	☐ No

SHARED – CONFIDENTIAL / خاص – مشترك

Section 2: Attitudes regarding the prevention and treatment of metabolic syndrome.

Attitude items	Disagree	Neutral	Agree
1. Early identification of patients with metabolic syndrome is crucial for addressing their diverse risk factors effectively.	☐	☐	☐
2. There is a strong association between obesity and leading sedentary lifestyles, both of which are closely linked to metabolic syndrome.	☐	☐	☐
3. There is a pressing need for significant changes in the habits of the general public in the United Arab Emirates.	☐	☐	☐
4. The population in the United Arab Emirates is cognizant of the correlation between metabolic syndrome and an increased predisposition to cardiovascular diseases and other non-communicable diseases (NCDs).	☐	☐	☐
5. Metabolic syndrome is prevalent in the UAE, and its incidence is on the rise.	☐	☐	☐

Section 3: Practices regarding the prevention and treatment of metabolic syndrome.

Practice items	Yes	No
Advising hypertension patients to limit their salt intake.	☐	☐
Counseling patients on the benefits of consuming more soluble fiber.	☐	☐
Referring patients to appropriate healthcare professionals or clinics as needed	☐	☐
Encouraging patients to increase their physical activity levels.	☐	☐
Selling home blood pressure and glucose monitoring devices to patients.	☐	☐
Providing dietary advice aimed at lowering cholesterol levels by reducing intake of cholesterol and saturated fat.	☐	☐
Maintaining comprehensive records of all patient care services provided.	☐	☐
Recommending a low-calorie diet to aid in weight reduction for patients.	☐	☐
Educating patients about the importance of regular monitoring of blood pressure, glycemic status, and weight, emphasizing the significance of achieving desired health outcomes.	☐	☐
Counseling patients to adhere to their prescribed treatment regimens.	☐	☐
Monitoring and evaluating patient responses to treatment plans.	☐	☐
Advising patients on over-the-counter medications and self-care techniques for managing components of metabolic syndrome	☐	☐
Advising patients to eat more vegetables that are derived from plants.	☐	☐
Offering guidance and support to patients in quitting smoking	☐	☐
